# The PK/PD Interactions of Doxycycline against *Mycoplasma gallisepticum*

**DOI:** 10.3389/fmicb.2016.00653

**Published:** 2016-05-04

**Authors:** Nan Zhang, Xiaoyan Gu, Xiaomei Ye, Xun Wu, Bingxu Zhang, Longfei Zhang, Xiangguang Shen, Hongxia Jiang, Huanzhong Ding

**Affiliations:** Guangdong Key Laboratory for Veterinary Drug Development and Safety evaluation, South China Agricultural UniversityGuangzhou, China

**Keywords:** *Mycoplasma gallisepticum*, doxycycline, PK/PD, *in vitro* model, kill rate

## Abstract

*Mycoplasma gallisepticum* is one of the most important pathogens that cause chronic respiratory disease in chicken. This study investigated the antibacterial activity of doxycycline against *M. gallisepticum* strain S6. In static time–killing studies with constant antibiotic concentrations [0–64 minimum inhibitory concentration (MIC)], *M. gallisepticum* colonies were quantified and kill rates were calculated to estimate the drug effect. The half-life of doxycycline in chicken was 6.51 ± 0.63 h. An *in vitro* dynamic model (the drug concentrations are fluctuant) was also established and two half-lives of 6.51 and 12 h were simulated. The samples were collected for drug concentration determination and viable counting of *M. gallisepticum*. In static time–killing studies, doxycycline produced a maximum antimycoplasmal effect of 5.62log_10_ (CFU/mL) reduction and the maximum kill rate was 0.11 h^−1^. In the *in vitro* dynamic model, doxycycline had a mycoplasmacidal activity in the two regimens, and the maximum antimycoplasmal effects were 4.1 and 4.75log_10_ (CFU/mL) reduction, respectively. Furthermore, the cumulative percentage of time over a 48-h period that the drug concentration exceeds the MIC (%*T* > MIC) was the pharmacokinetic–pharmacodynamic index that best correlated with antimicrobial efficacy (*R*^2^ = 0.986, compared with 0.897 for the peak level divided by the MIC and 0.953 for the area under the concentration–time curve over 48 h divided by the MIC). The estimated %*T* > MIC values for 0log_10_ (CFU/mL) reduction, 2log10 (CFU/mL) reduction and 3log_10_ (CFU/mL) reduction were 32.48, 45.68, and 54.36%, respectively, during 48 h treatment period of doxycycline. In conclusion, doxycycline shows excellent effectiveness and time-dependent characteristics against *M. gallisepticum* strain S6 *in vitro*. Additionally, these results will guide optimal dosing strategies of doxycycline in *M. gallisepticum* infection.

## Introduction

*Mycoplasma gallisepticum* belongs to the class *Mollicutes*, order *Mycoplasmatales*, family *Mycoplasmataceae* which is characterized by lacking a cell wall (Athamna et al., 1997; Uilenberg et al., 2006). Similar to many other pathogenic mycoplasmas, this microorganism colonizes its host via the mucosal surface of the respiratory tract and the most paramount process to develop an infection is the adhesion of *M. gallisepticum* to its host target cell initially. Besides, *M. gallisepticum* colonizes the respiratory tract usually as a risk factor for subsequent systemic infection, such as salpingitis and arthritis (Winner et al., 2000; Nascimento et al., 2005). This infectious disease spreads vertically through infected eggs and horizontally by close contact, which result in worldwide prevalence of *M. gallisepticum* infection. The disease brings serious economic loss to farmers. In an earlier published report, *M. gallisepticum* infection caused reduction in egg production, weight gain, and feed conversion efficiency of 10–20%, and an increase in embryo mortality and chick mortality of 5–10% (Kleven, 1990). Antimicrobial chemotherapy plays a significant role in treating infections of *M. gallisepticum*. However, mycoplasma has been regarded as a kind of fastidious and slowly growing microorganism. *Mycoplasma* requires appropriate growth media and culturing condition, so research on mycoplasma is difficult in many instances. Quantitative cultures have been established with color changing unit (CCU) or CFU determination by serial dilution and plating on appropriate agar (Stemke and Robertson, 1982). Even though viable count estimation is complicated by strict nutritional conditions of *M. gallisepticum* cell, the CFU determination is an accurate measure for quantifying the number of *M. gallisepticum* clone, and resistant *M. gallisepticum* stains could be selected from the agar plate.

In the past decades, there were a few reports concerning PK/PD integration of antimicrobials against mycoplasmas. Mitchell et al. (2012, 2013a,b) have evaluated the antimicrobial activity against *M. mycoides subspecies mycoides Small Colony* by *in vitro* dilution PK/PD model. Xiao et al. (2015a,b) have studied the PK/PD relationship of valnemulin against *M. gallisepticum* by *ex vivo* model and *in vivo* model, with a real-time PCR (RT-PCR) method to detect *M. gallisepticum*. However, a more reasonable model and more accurate viable count estimation should be applied to explore the relationships between antimicrobials and mycoplasmas.

A multitude of agents inhibiting protein synthesis have been demonstrated to be efficacious against *M. gallisepticum*, including tetracyclines, macrolides, quinolones, and tiamulin (Cooper et al., 1993; Bradbury et al., 1994; Gerchman et al., 2008; Zakeri and Kashefi, 2011). Doxycycline is a semi-synthetic bacteriostatic tetracycline whose pharmacokinetic property is superior to older tetracyclines (Soliman et al., 2015). In this study, the *in vitro* dynamic model to simulate the pharmacokinetic profiles of doxycycline in chickens was established on the basis of the *in vitro* pharmacokinetic/pharmacodynamic model used to evaluate the properties of antifungal drugs against *Aspergillus* species (Meletiadis et al., 2012). Besides, quantitative culture was performed by counting colonies on ager plate to investigate the PK/PD interaction of doxycycline against *M. gallisepticum.* Moreover, because the growth characteristic of *M. gallisepticum* is significantly different from other microorganisms, the kill rate was introduced so as to evaluate the pharmacodynamics of doxycycline in the time–killing study. The purpose of this study was to characterize the activity of doxycycline against S6 strain of *M. gallisepticum* by *in vitro* static time–killing studies preliminarily. Then PK–PD index that best correlated with antimicrobial efficacy was analyzed. The results of this study could provide guidance for dosing regimen design in the clinical treatment of *M. gallisepticum* infection.

## Materials and Methods

### Materials

The *M. gallisepticum* standard strain S6 and doxycycline standard were obtained from China Institute of Veterinary Drugs Control, Beijing. *M. gallisepticum* artificial medium base was purchased from Qingdao Hope Biological Technology. Sterile pig serum was provided by Guangzhou Ruite Biological Technology. Nicotinamide adenine dinucleotide (NADH) and cysteine were purchased from Guangzhou prob information Technology. Doxycycline powder was supplied by Guangdong Dahuanong Animal Health Products. Fresh solutions of doxycycline were prepared in ultrapure water at 3200 mg/L on the day of the experiment.

Twelve Sanhuang broiler chickens were kindly supplied by from the Guangdong Academy of Agricultural Sciences. They were housed in cages and supplied with water *ad libitum*. Chickens were fed on a balanced ration free from any antibiotic drugs before starting experiments to ensure complete clearance of their bodies from any drug residues. All husbandry practices and experimental operations were performed with full consideration of animal welfare. Research ethical approval was granted by the South China Agriculture University Animal Ethics Committee (2015-A027).

### Determination of Minimum Inhibitory Concentration (MIC)

Minimum inhibitory concentration of doxycycline against *M. gallisepticum* strain S6 was determined by using a modified MIC assay method, as described by Tanner and Wu (1992). Briefly, the final concentration of doxycycline ranging from 0.03125 to 16 mg/L in each well and serial twofold dilutions of doxycycline were performed. The exponential-phase culture was diluted with medium to the desired inoculum size between 10^5^ and 10^7^ CFU/mL. Serial twofold dilutions of drug were applied, containing 0.1 mL of each compound dilution and supplemented with 0.1 mL of *M. gallisepticum* inoculum from the first to the ninth column. 0.2 mL of sterile *M. gallisepticum* broth at pH 7.8 was added to well number 10, as a sterility control. Moreover, growth control (*M. gallisepticum* inoculum in absence of antimicrobials), end-point control (blank medium at pH 6.8) were also included. The *M. gallisepticum* count of the inoculum must be tested simultaneously with the MIC determination to confirm the inoculum that ranging from 10^5^ to 10^7^ CFU/mL. After being sealed with gas permeable film, plates were cultured in a 37°C, 5% CO_2_ humidified incubator. The MIC was determined as the minimal concentration of doxycycline that resulted in no change in color. Changes of color were monitored three times a day until the color of the growth control was same as that of the end-point control. All experiments were performed in triplicates.

### Exposure to Constant Antibiotic Concentrations

Eight clinically achievable concentrations of doxycycline were used (0, 0.5, 1, 2, 4, 8, 16, 32, and 64 MIC). 0.4 mL of the suspension in exponential phase was added into a 7-mL penicillin bottle to obtain the initial titer whose inoculum size was approximately 10^7^ CFU/mL, each containing 0.1 mL of drug solution at 40 times the target concentration and 3.5 mL blank medium. These penicillin bottles were cultured for 48 h at 37°C and aliquots of 100 μL of each culture were removed at 0, 2, 12, 15, 18, 24, 26, 36, 38, and 48 h to characterize the effect of various concentrations of doxycycline on the total *M. gallisepticum* population. Growth control (*M. gallisepticum* culture in the absence of antimicrobials) and sterility control (4 ml of *M. gallisepticum* medium at the pH 7.8 was added into the penicillin bottle) were included. The viable counts of *M. gallisepticum* were quantified by 10-fold serial dilutions of the samples, then 10 μl of each *M. gallisepticum* dilutions (in triplicate or more for greater accuracy) were transferred to the surface of *M. gallisepticum* agar plates which were freshly prepared and dried. Plates were incubated at least 7 days in a 37°C, 5% CO_2_ humidified incubator. The theoretical lower limit of detection was 100 CFU/mL and time–killing studies were conducted on different days to ensure reproducibility of the data.

### Time–Killing Curve Fitting and Analysis

The change in the kill rate over time has also been described as the result of drug effects on a heterogeneous population. Once growth rates reduced, the susceptibility reduced (Nielsen et al., 2007). Our group has taken the following approach to obtain the kill rate. Firstly, time–killing curve were depicted by plotting log_10_ (CFU/mL) against time (h) with different concentrations of doxycycline. Additionally, kill rate was reflected by the slope of the curve at the given time interval (0∼24, 0∼36, 0∼48, 2∼24, 2∼36, 2∼48, and 12∼48 h). As a result, seven kill rates were obtained from each concentration. Besides, the kill rates were also calculated from 2 h and 12 h because that delayed growth of *M. gallisepticum* was observed by transferring to the culture vessel. Finally, linear regression was used to calculate the slope for each time interval, and then the kill rate (the slop value) and the doxycycline concentrations were fitted to the *E*_max_ model employing WinNonlin (version 6.1, Pharsight Corporation, Mountain View, Sunnyvale, CA, USA). The *E*_max_ model could be described by the following equation:

(1) $$\begin{array}{cc} E=\frac{E_{\text{max}}\times C_{e}^{N}}{EC_{50}^{N}+C_{e}^{N}} & \end{array}$$

Where *E* is the kill rate, *E*_max_ is the maximum kill rate in a certain period of time, *C*_e_ is the doxycycline concentration, *N* is the Hill coefficient that describes the steepness of the kill rate-effect curve. *EC*_50_ is the doxycycline concentration which produced 50% of the maximum kill rate. The correlation coefficient *R*^2^ represented the strength and direction of relationship between the experimental data (observed values) and the *E*_max_ model (predicted values) on a scatterplot. Because the kill rates were obtained from the different time periods, seven scatterplots could be depicted. The value *R*^2^ quantifies goodness of fit, thus the higher values indicated that the data fits the model better in the corresponding time period.

### Pharmacokinetics of Doxycycline in Chicken

Doxycycline was administered intravenously at a single dose of 10 mg/kg bodyweight (BW) to 12 broiler chickens. The mean body weight (±*SD*) of the chicken was 1.53 ± 0.31 kg. Blood samples were taken from the left brachial wing vein of each chicken just before and at 0.08, 0.17, 0.25, 0.5, 1, 2, 4, 6, 8, 10, 12, 24, 36, and 48 h post-antibiotic injection. Blood samples were centrifuged at 3000 *g* for 10 min and plasma were collected and stored at −20°C until assayed for doxycycline. The concentration of doxycycline was determined by high-pressure liquid chromatography–tandem mass spectrometry HPLC–MS/MS as described elsewhere (Patel et al., 2011). The PK data of doxycycline were analyzed by WinNonlin software.

### Exposure to Changing Antibiotic Concentrations

The *in vitro* pharmacodynamic model has been described in detail previously (Meletiadis et al., 2012). Briefly, a modified three-necked flask containing 320 mL medium (external compartment [EC]) could be more conducive to sampling and also reduce the risk of contamination. Three necks of the flask served as inlet tubing, sampling tube, and outlet tubing, respectively. The sampling tube consisted of a rubber stopper, two elongated syringe needles, and two 0.22-μm nylon filters.

The intravenous formulation and long-acting parenteral formulation were simulated by two half-lives of doxycycline which were 6.51 and 12 h. At time zero, doxycycline was injected into both the EC and the IC (internal compartment) simultaneously, which resulted in the initial drug concentration from 1 to 64 MIC of doxycycline. Meanwhile, the IC was inoculated with 10 mL medium containing approximately 10^7^ CFU/mL of *M. gallisepticum*. Then the drug-free medium was pumped continuously into the EC from the reservoir to dilute the drug, and a second pump guaranteed the discharging. The drug concentrations conformed to the first order model: *C* = *C*_0_e^−kt^, *C*_0_ is the initial doxycycline concentration, *C* is the doxycycline concentration at time *t*, *k* is the rate of elimination and *t* is the time that has elapsed since the addition of doxycycline.

The experiment was conducted for 48 h, the EC was placed in a water jacket to keep the temperature constant at 37°C and a magnetic stirrer was used to keep the media homogeneous. Samples were collected from the EC (2 mL) and IC (0.1 mL) simultaneously at 2, 12, 15, 18, 21, 24, 36, and 48 h after injection during the experiment for drug concentration determination and viable counting of *M. gallisepticum*. All experiments were performed in triplicates.

### Pharmacokinetic Analysis

Serial samples were obtained from the EC and stored at −20°C until being assayed. The concentration of doxycycline was determined by using a previously described method (high-pressure liquid chromatography–tandem mass spectrometry HPLC–MS/MS, Agilent Technologies, USA; Tarapoulouzi et al., 2013). The mobile phase consisted of solution A (water with 0.1% formic acid, V/V) and solution B (acetonitrile) at 0.25 mL/min flow rate. The gradient elution was: 0–1 min, 90% A; 1–4 min, 70% A; 4–5 min, 50% A; 5–13 min, 90% A. The injection volume was 5 μL. The half-life for each regimens, area under the concentration versus time curve (AUC_0-48h_), mean residence time, volume of distribution, and total body clearance of doxycycline were calculated from the concentration–time plot by using a one-compartment pharmacokinetic model for intravenous bolus administration. AUC_0-48h_ was calculated because the pharmacokinetic parameter values were determined after a single dose and the period was 48 h.

### Pharmacokinetic–Pharmacodynamic Integration and Modeling

Specific PK/PD indices (*T* > MIC, *C*_max_/MIC, AUC_0-48h_/MIC) have been identified which are of major importance for the assessment of antimicrobial activity against *M. gallisepticum*, reducing the emergence of resistance and establishing a rational dosage regimen. For PK–PD integration, the surrogate indices were calculated according to a one compartmental analysis by WinNonlin. The software was employed to fit data to a sigmoid *E*_max_ model which was described by the formula as follows.

(2) $$\begin{array}{cc} E=E_{0}-\frac{{\text{I}}_{\text{max}}\times C_{e}^{N}}{\text{I}C_{50}^{N}+C_{e}^{N}} & \end{array}$$

Where *E* is the antimycoplasmal effect, *E*_0_ is the change in log_10_ (CFU/mL) of control sample (absence of doxycycline) after 48 h incubation. I_max_ is the maximum antimycoplasmal effect determined as log_10_ (CFU/mL) reduction in samples incubated with doxycycline between time 0 and 48 h. I*C*_50_ is the drug concentration that gives rise to 50% of the maximum antimycoplasmal response. *C*_e_ is the PK/PD indices (*C*_max_/MIC, AUC_0-48h_/MIC and *T* > MIC) in the effect compartment, while *N* is the Hill coefficient that describes the steepness of the PK/PD indices-effect curve and *R*^2^ were calculated for each assay. Values for *E* between 0 and −3log_10_ (CFU/mL) reduction were considered to be of mycoplasmastasis activity, whereas values less than or equal to −3log_10_ (CFU/ml) denoted mycoplasmacidal activity.

## Results

### Minimum Inhibitory Concentration

The MIC of doxycycline for *M. gallisepticum* strains S6 was 0.125 mg/L for the three different inoculum sizes (10^5^, 10^6^, and 10^7^ CFU/mL). No growth of *M. gallisepticum* was observed in the sterilizing control, end-point control, while growth was observed in the positive control, and validating the experiments.

### Static Time–Killing Curves and Analysis

**FIGURE 1:**
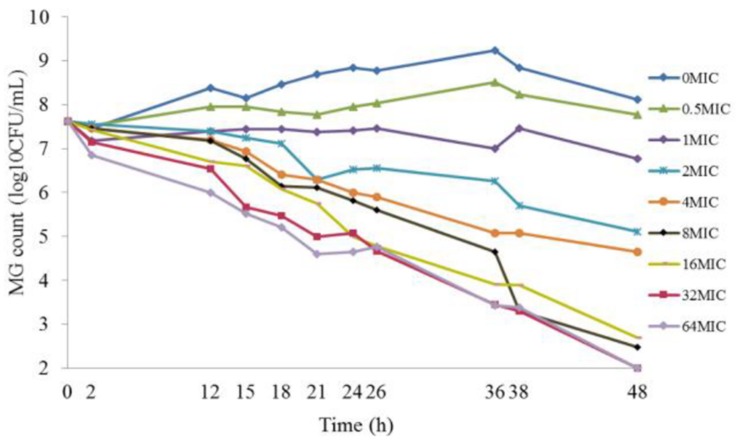
**Time–killing studies of doxycycline (as multiples of MIC) against ***Mycoplasma gallisepticum*** with constant concentrations**. Data are presented as geometric means based on triplicates.

The results of the static time–killing study are presented as dots in **Figure 1**. These doxycycline doses produced marked reductions in organism burden ranging from 0.84 to 5.62log_10_ (CFU/mL) reduction with *M. gallisepticum*, compared with the starting inoculum. At low concentration of doxycycline (0.5 MIC), *M. gallisepticum* counts were slight increased by 0.15log_10_ (CFU/mL). Inhibition of *M. gallisepticum* growth was observed in 1∼4 MIC, decreased by 0.84∼2.97log_10_ (CFU/mL) during the experimental process. There was mycoplasmacidal activity, more than 4log_10_ (CFU/mL) reduction, when *M. gallisepticum* was exposed to high concentration of doxycycline at 8∼64 MIC. Actually, the 32 MIC has already achieved the maximal effect, showing 5.62log_10_ (CFU/mL) reduction during 48 h. As the antibiotic concentration increased (0.5∼8 MIC), the antimycoplasmal activity gradually increased. Nonetheless, there was no significant difference in the decline number of *M. gallisepticum* colonies between 8 and 64 MIC. Overall, the antimycoplasmal effect appeared not to be always dependent on concentration.

**FIGURE 2:**
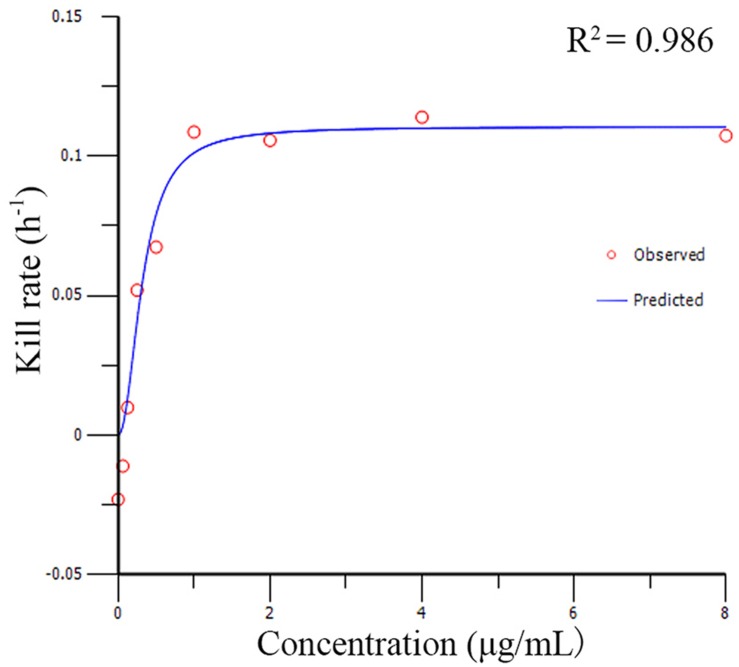
**The best-fit curve obtained from the ***E***_max_ model of ***M. gallisepticum*** exposed to doxycycline between 0 and 48 h**. *R*^2^ is the correlation coefficient.

The relationships between the kill rate and drug concentations is represented graphically in **Figure 2** (the time interval was 0∼48 h). All pharmacodynamic parameters are obtained from the *E*_max_ model with variable slopes and the data was satisfactorily fitted to the model (the mean *R*^2^ was 0.961 ± 0.026). The maximum *R*^2^ for kill rate-concentration curve was 0.986 and the maximum kill rate was 0.11 h^−1^ from the period of 0∼48 h. The kill rate profile of doxycycline against *M. gallisepticum* manifested that when the concentration exceeded 8 MIC, the kill rates seemed to be a plateau after a rapid rise phase. The results of model analysis also confirmed that the growth rate for *M. gallisepticum* was relatively low (0.02 h^−1^). The relationship between concentration and kill rate was fitted by *E*_max_ model, and the obtained parameters of *E*_max_, EC_50_, Hill coefficient are presented in **Table 1**.

**Table 1:** The kill rate parameter estimation derived from the *E*_max_ model which fitted to *in vitro* static time–killng assay data.

Time (h)	*E*_max_ (h^−1^)	EC_50_ (μg/mL)	Hill’s slope	*R*^2^
0–24	0.12	0.02	1.45	0.958
0–36	0.11	0.38	2.21	0.971
0–48	0.11	0.32	2.11	0.986
2–24	0.1	0.33	2.21	0.955
2–36	0.1	0.32	2.47	0.97
2–48	0.11	0.31	2.83	0.907
12–48	0.12	0.28	2.2	0.977

### Pharmacokinetics Analysis in Chicken

**FIGURE 3:**
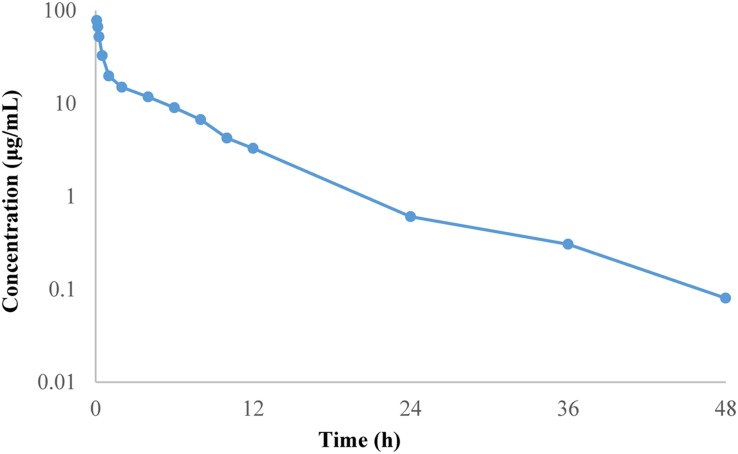
**Mean plasma concentration–time profile of doxycycline following intravenous administration at a single dose of 10 mg/kg BW to chickens**.

The mean plasma concentration–time curve of doxycycline in chickens are shown in **Figure 3**. The PK parameters are summarized in **Table 2**. The half-life was 6.51 ± 0.63 h, mean residence time was 6.21 ± 0.42 h, volume of distribution was 0.54 ± 0.17 L/kg and total body clearance of doxycycline was 0.06 ± 0.12 L/h kg. AUC_0-24h_ was 164.40 ± 4.78 μg h/mL.

**Table 2:** Pharmacokinetic parameters of doxycycline in plasma following intravenous administration at a single dose of 10 mg/kg BW to 12 chickens.

Parameters (units)	Mean ± standard deviations (*SD*)
AUC_0-24h_ (μg h/mL)	164.40 ± 4.78
MRT (h)	6.21 ± 0.42
CL (L/h⋅kg)	0.06 ± 0.12
V (L/kg)	0.54 ± 0.17
T_1/2_(h)	6.51 ± 0.63

*AUC_0-24 h_, 24 h area under plasma concentration–time curve; MRT, mean residence time; CL, total body clearance; V, volume of distribution; T_1/2_, elimination half-life.*

### Pharmacokinetics in *In Vitro* Dynamic Model

**FIGURE 4:**
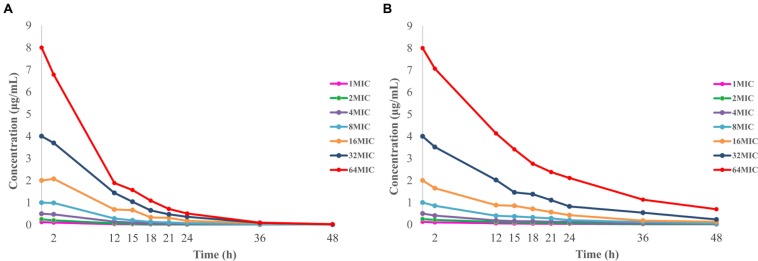
**Concentration–time curves of doxycycline in the *in vitro* dynamic model simulating two half-lives of 6.51 h (A) and 12 h (B)**.

The limit of detection and limit of quantification for the developed method was 5 and 10 ppb (ng/mL), respectively. A calibrated curve was constructed by adding a known amount of doxycycline to blank *M. gallisepticum* culture over concentrations ranging from 0.005 to 0.5 μg/mL (*R*^2^ > 0.999). The within-run relative standard deviations (RSDs; *n* = 6) for doxycycline quality control concentrations of 0.1, 0.02, and 0.01 μg/mL were 2.02, 1.43, and 2.37%. The between-run RSDs for the same quality control concentrations (*n* = 18) were 2.32, 1.70, and 2.83%. The recoveries of doxycycline in drug-free medium were 84.54 ± 0.68% (mean ± standard deviation, *SD*, *n* = 6). The time–concentration curves for *in vitro* dynamic model are shown in **Figure 4**. Half-lives were calculated to be 6.19 ± 0.43 and 11.93 ± 0.74 h, and mean residence times were 8.29 ± 0.36 h and 17.03 ± 0.41 h, respectively. Volume of distribution were 0.90 ± 0.10 and 0.99 ± 0.20 L, and total body clearance were 0.10 ± 0.01 and 0.06 ± 0.01 L/h, according to the initial experimental design of 6.51 and 12 h half-life, respectively.

### Dynamic Time–Killing Curves and Analysis

**FIGURE 5:**
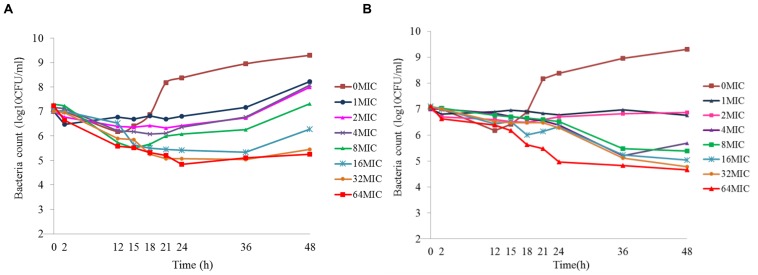
**Dynamic time–killing curves were depicted at different concentrations of doxycycline for the two simulated regimens**. The mean half-lives were 6.19 ± 0.43 h and **(A)** and 11.93 ± 0.74 h **(B)**. Data points represent geometric means of three experiments.

**Figure 5** shows the effect of antibiotic against *M. gallisepticum* at different concentrations of two simulated regimens whose half-lives were 6.51 and 12 h. Escalating doses of doxycycline resulted in marked killing of *M. gallisepticum*. Regrowth was observed in all regimens except one that simulating the *C*_0_ of doxycycline was 64 MIC and half-life was 11.93 h. Doxycycline was mycoplasmacidal activity in the two regimens whose *I*_max_ were 4.1 and 4.75log10 (CFU/mL) reduction. Since the EC was continuously diluted by drug-free medium, the curves tended to gently decline. When the drug concentration dropped below the MIC, the regrowth of the *M. gallisepticum* was detected promptly. On the whole, the **Figure 5** illustrated that the potency of doxycycline with long half-life was superior to the short half-life.

### Pharmacokinetic–Pharmacodynamic Modeling and Analysis

**FIGURE 6:**
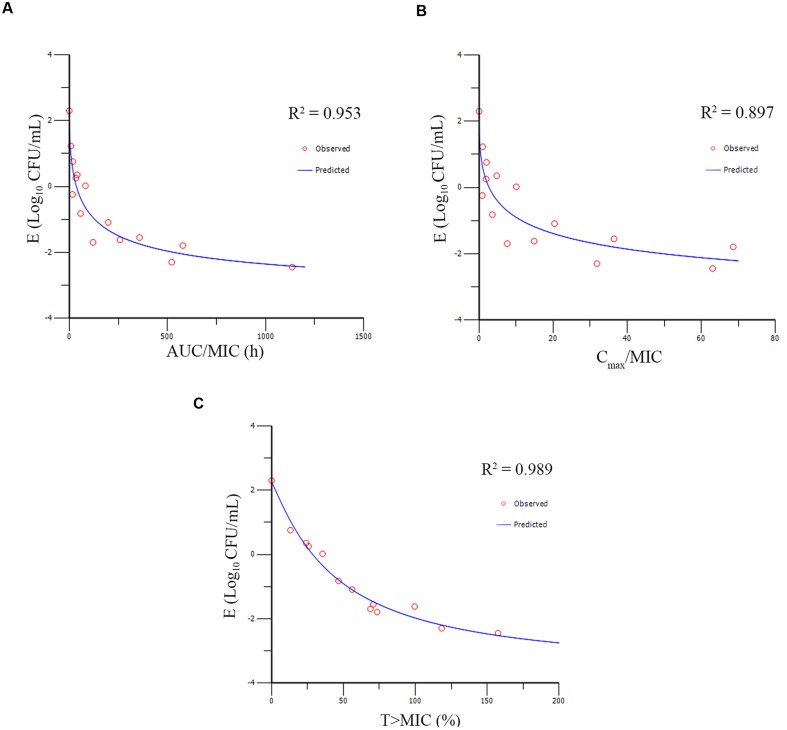
*****E***_max_ relationships for the three PK/PD parameters versus antimycoplasmal effect. (A)** AUC_o-48h_/MIC-antimycoplasmal effect curve; **(B)** *C*_max_/MIC- antimycoplasmal effect curve; **(C)** *T* > MIC-antimycoplasmal effect curve. *R*^2^ is the correlation coefficient.

The PK/PD parameters (AUC_0-48h_/MIC; *C*_max_/MIC; *T* > MIC) derived from dynamic time–killing assays and antimycoplasmal effect are expatiated in **Figure 6**. The strongest relationship was observed when antimicrobial effect was correlated with the %*T* > MIC, with an *R*^2^ value of 0.989 (AUC_0-48h_/MIC *R*^2^ = 0.953; *C*_max_/MIC *R*^2^ = 0.897). The above studies indicated that antimycoplasmal activity depended on the percentage of time above the MIC and suggested that efficacy was driven by the %*T* > MIC parameter. The relationship between antibacterial efficacy and the PK/PD parameters was assessed by using the sigmoid *E*_max_ model, and the obtained parameters of E_0_, *I*_max_, and IC_50_, and the Hill coefficient are presented in **Table 3**. The estimated %*T* > MIC values for 0log_10_ (CFU/mL) reduction, 2log10 (CFU/mL) reduction and 3log_10_ (CFU/mL) reduction were 32.48, 45.68, and 54.36%, respectively, during 48 h treatment period of doxycycline.

**Table 3:** The PK/PD Parameter estimation, and the data are derived from *E*_max_ model.

PK/PD parameter	*I*_max_ (log_10_ CFU/mL)	IC_50_ (μg/mL)	*E*_0_ (log_10_ CFU/mL)	Hill’s slope	*R*^2^
AUC_0-48h_/MIC (h)	5.82	79.12	2.30	0.55	0.953
*C*_max_/MIC	6.49	10.90	2.28	0.44	0.897
*T* > MIC (%)	5.93	44.39	2.25	1.12	0.989

## Discussion

*Mycoplasma gallisepticum* is one of the most economically significant pathogens of poultry, and has a world-wide distribution. In common with other mycoplasmas, *M. gallisepticum* is the smallest known self-replicating organism and lacks a cell. These characteristics of mycoplasmas were also reflected in a high degree of interdependence between mycoplasmas and the host animal, and in the fastidious nature of the organism *in vitro* (Levisohn and Kleven, 2000). So far, there is limited information available on the PK/PD interactions of antibacterial agents against mycoplasmas. Mitchell et al. (2013b) have evaluated the antimicrobial activity against *M. mycoides subspecies mycoides Small Colony* in *in vitro* dilution PK/PD model. They have evaluated a series of antimicrobials activity *in vitro*, but in the *in vitro* model, the dilution was simulated by increasing volume. A continuous dilution and an outlet to mimic elimination of drug have not been offered by this model. Whereas our dynamic model not only provided the continuous flow without increasing volume, but also eliminated the agent through an outlet; Xiao et al. (2015a,b) have studied the PK/PD relationship of valnemulin against *M. gallisepticum* by *ex vivo* model and *in vivo* model with the target species. The dosage regimen for valnemulin against *M. gallisepticum* based on PK/PD modeling and a real-time PCR (RT-PCR) method quantitative detection of *M. gallisepticum* were provided. However, it is difficult for CCU method to quantitate the number of *M. gallisepticum* accurately, for the reason that *M. gallisepticum* belongs to the slow growing microorganism. The results can only be recorded at least 7 days later until the color changed from red to orange or yellow, while the color of liquid medium may also be changed by acidification of carbon dioxide over time, which may lead to some confusion. Although the RT-PCR methodology gave result quickly and specifically, this detection method was performed indirectly and may be impeded by other organisms or dead subculture. The CFU quantitative detection adopted by us in this study is accurate because the *M. gallisepticum* colony is recorded from inverted microscope directly and the resistant mutant could be selected from the agar plate. Arai et al. (1993) have studied various agents and treatment regimens for experimental *M. pneumonia*. The rat infection model was utilized, and CFU of *M. pneumonia* in lung and bronchoalveolar lavage samples were quantitated as well (Ishida et al., 1994; Kaku et al., 1994). Nevertheless, the drug concentrations and log_10_ (CFU/mL) reduction have not been fitted by PK/PD modeling that could provide a foundation for the application of antibacterial agents against *M. pneumonia* infections. In addition, in the two aforementioned *in vivo* models, changes in the amount of bacteria during the experiment were not recorded continuously, while continuous colony counting was performed in our dynamic *in vitro* model. In this study, we strived to investigate the PK/PD interactions of doxycycline against *M. gallisepticum* in *in vitro* model. Available information was provided by our results, which could be used to support dose selection in a more rational manner.

The MIC of doxycycline against *M. gallisepticum* strain S6 was 0.125 mg/L. In the determination of MIC, the final inoculum size of *M. gallisepticum* in each well was between 10^5^ and 10^7^ CFU/mL. Although the recommended turbidity of MIC testing against veterinary mycoplasma species was between 10^3^ and 10^5^ CFU/mL (Hannan, 2000), the turbidity of the growth phase of the mycoplasmas seemed less important (Whithear et al., 1983). The inoculum size was replaced by current method and the same results were obtained for the inoculum size between 10^5^ and 10^7^ CFU/mL. There were two primary reasons for choosing the high inoculum. First, the population of *M. gallisepticum* was consistent with the *in vitro* dynamic experiment where the initial inoculum was approximately 10^7^ CFU/mL. Second, the high inoculum was applied to simulate the *M. gallisepticum* density in severe infection, and may allow a resistant subpopulation(s) to be present, as the real situation in infection site. Furthermore, the MIC of doxycycline against *M. gallisepticum* strain S6 was similar to the previous report in which the final concentration of the *M. gallisepticum* organisms was approximately 10^4^ CFU/ml (Pakpinyo and Sasipreeyajan, 2007).

It has been reported that oxytetracycline lacked mycoplasmacidal activity against clinical strains of *M. mycoides subspecies mycoides Small Colony* when the assays were conducted for 12 and 24 h under constant concentration (Mitchell et al., 2012, 2013b). However, the present study showed that the *M. gallisepticum* achieved mycoplasmacidal activity against *M. gallisepticum* after 48 h exposure. One explanation may be that a delay in the growth of *M. gallisepticum* was observed, possibly related to the fact that *M. gallisepticum* was not instantaneously in their logarithmic growth phase when transferred to the culture vessel. Another may be that doxycycline is a slow-acting bacteriostatic agent and associated with reversible inhibition of protein synthesis. So it may be more reasonable to study the antimycoplasmal activity in a longer time periods (48 h).

One novel, crucial step for the kinetic model is the establishment of *E*_max_ model between kill rate and antimicrobial agent concentration. In some cases, only the initial slope of the killing curves were employed for the pharmacodynamic parameters (Regoes et al., 2004; Mouton and Vinks, 2005; Treyaprasert et al., 2007). Although this approach is propitious to microorganism that grow quickly, it seemed to be inaccurate when the initial slope is not log-linear. However, our results illustrated that the *M. gallisepticum* grew slowly and the growth rate was 0.02 h^−1^. Accordingly, in our study, it was improper to estimate the pharmacodynamic parameters with the initial slope. In this study, WinNonlin was employed to fit the drug concentration and the kill rate (obtained from different intervals). There was no significant difference in the estimated *R*^2^ value from the different intervals, and the maximum *R*^2^ value came from the longest time period 0∼48 h which was our experimental cycle. It was desirable to estimate the overall effect of antibiotic agent on *M. gallisepticum* through the longest observation period. Furthermore, for the purpose of assessing the killing profiles in different concentration, we modeled the experimental data mathematically by kill rate to provide an objective and quantitative evaluation. The kill rate of drug was influenced by time exposed to the microorganism or the various concentrations of drug. Hence the kill rate was divided into time-dependent and concentration-dependent (Craig, 2002). **Figure 2** suggested that the kill rate was time-dependent effect because the kill rate remained constant when the concentration exceeded 8 MIC. Some investigators argue that time-dependent drugs do not lead to faster killing, whereas concentration-dependent drugs do when the antibiotic concentration exceeded 4 MIC. However, others asserted that, based on visual inspection of killing curve data, it was controversial that the threshold value was 4 MIC (Craig, 1998; Ambrose, 2007).

Our research suggested that doxycycline exhibited time-dependent killing in *in vitro* model. In the past decades, there is only one study that identified PK/PD index of doxycycline. The result has showed that doxycycline was time-dependent kinetic at low serum concentrations but concentration-dependent kinetic at high serum concentrations against *Escherichia coli*, *Staphylococcus aureus*, *Pasteurella multocida*, and *Streptococcus pneumoniae* (Cunha et al., 2000). However, our results showed that doxycycline was time-dependent characteristics against *M. gallisepticum*, and this difference may be caused by different target microorganism. This phenomenon had once been officially reported by other people (Tam et al., 2006), one of his study found that gentamicin exhibited time-dependent killing profiles against *Staphylococcus aureus* but concentration-dependent killing profiles against *Pseudomonas aeruginosa*.

In *in vitro* model, it is possible to have full control of the drug profile within the system and samples could be continuously collected to monitor the growth and dying behavior of the bacteria. The PK/PD relationship represents a key link between optimal drug dosing and clinical outcomes, which is crucial to optimize animal outcomes and reduce the risk of resistance. Since the drug concentrations in time–killing studies were constant over time, the clinical relevance of the results might not be very evident. Therefore, an *in vitro* dynamic infection model was expected to provide further clinical insights. The model was developed by Meletiadis et al. (2012) and it was soon be used in additional studies by the same research group (Al-Saigh et al., 2012, 2013). The merits of the dialysis-tube infection model were extensive. For instance, a steady state of nutrient and *M. gallisepticum* were obtained in these open systems, without the bacteria loss and penetrating into the reservoir, which could falsifies the results. Moreover, the *in vitro* dynamic model was modified and showed great advantage over the other *in vitro* PK–PD models for *M. gallisepticum*, as it considered the sterile environment and special needs of the investigation. **Figure 5** showed that better antimycoplasmal effect was attained with the longer half-life. The explanation might be that the drug concentration decreased more slowly, leading to a longer lasting effect with the half-life of 12 h. The regrowth of *M. gallisepticum* was observed in the dynamic model. Two main empirical points of view were speculated for the mechanism of regrowth. Primarily, the total *M. gallisepticum* population consisted of a few distinct and discrete *M. gallisepticum* populations, and the preexisting resistant subpopulation may be selected to prosper and replaced the entire population when challenged with an antibiotic. Consequently, regrowth and the emergence of resistance as a gradual process evolved over time. In addition, the regrowth of *M. gallisepticum* after initial decline of the mycoplasma population was due to an adaptation response.

With constant concentrations, doxycycline produced a maximum antimycoplasmal effect of 5.62log_10_ (CFU/mL) reduction. Moreover, kill rate studies not only provided accurate characterization of the effect of doxycycline than MIC alone, but also validated the time-dependent antimycoplasmal activity of doxycycline. The relationship between doxycycline concentration and kill rate was time-dependent characteristics and the maximum kill rate was 0.11 h^−1^. The *in vitro* dynamic time–killing assays also demonstrate that antimycoplasmal activity of doxycycline against *M. gallisepticum* was time-dependent characteristics and PK–PD parameters should be based on the %*T* > MIC (*R*^2^ = 0.986). However, time-dependent characteristics needs to be confirmed by further studies such as *in vivo* PK/PD model and fractionation schedules. These *in vitro* models will provide practical data that are much closer to the *in vivo* situation and may aid in the design of more reasonable treatment regimens.

## Author Contributions

Authors make substantial contributions to conception and design, and/or acquisition of data, and/or analysis, and interpretation of data: NZ, XG, HD, XW, XY, BZ, LZ, XS, and HJ. Authors participate in drafting the article or revising it critically for important intellectual content: NZ, XG, HD, XW, XY, BZ, LZ, XS, and HJ. Authors give final approval of the version to be submitted and any revised version: NZ, XG, HD, XW, XY, BZ, LZ, XS, and HJ. Agreement to be accountable for all aspects of the work in ensuring that questions related to the accuracy or integrity of any part: NZ, XG, HD, XW, XY, BZ, LZ, XS, and HJ.

## Conflict of Interest Statement

The authors declare that the research was conducted in the absence of any commercial or financial relationships that could be construed as a potential conflict of interest.
